# Usefulness of the Ranking Technique in the Microscopic Agglutination Test (MAT) to Predict the Most Likely Infecting Serogroup of *Leptospira*

**DOI:** 10.3389/fvets.2021.654034

**Published:** 2021-03-03

**Authors:** Israel Barbosa Guedes, Gisele Oliveira de Souza, Juliana Fernandes de Paula Castro, Matheus Burilli Cavalini, Antônio Francisco de Souza Filho, Marcos Bryan Heinemann

**Affiliations:** Departamento de Medicina Veterinária Preventiva e Saúde Animal, Faculdade de Medicina Veterinária e Zootecnia, Universidade de São Paulo, São Paulo, Brazil

**Keywords:** leptospirosis, serology, MAT, diagnostic, epidemiology

## Abstract

The microscopic agglutination test (MAT) used for the serological diagnosis of leptospirosis, as a robust and inexpensive method, is still the reality in many laboratories worldwide. Both the performance and the interpretation of the MAT vary from region to region, making standardization difficult. The prediction of the probable infecting serogroup using this test is indispensable for elucidating the epidemiology of the disease; however, in veterinary medicine, many studies consider any reaction detected with a titer of 100, which may ultimately overestimate some serogroups. Thus, the aim of this study was to evaluate the usefulness of the ranking technique for predicting the probable infecting serogroup identified by the MAT, eliminating cross reactions with other serogroups. *Leptospira* strains (12 samples) were inoculated in hamsters, and after 30 days, serology was performed by the MAT for these animals to confirm the infecting serogroup. Using the ranking technique, the probable infectious serogroup found with the MAT was the same as that in which the strains of inoculated leptospires belonged; additionally, the technique can be applied in epidemiological studies involving herds.

## Introduction

Leptospirosis is an emerging disease that affects various species of mammals, including humans. The disease is caused by pathogenic bacteria of the genus *Leptospira*, which encompass several serogroups that comprise a variety of serovars (1, 2). The great importance in knowing the predominant *Leptospira* serogroups circulating in a population is the contribution to elucidating the epidemiological chain of the disease, which influences the adoption of effective control measures (3).

The microscopic agglutination test (MAT) is considered a reference in the serological diagnosis of leptospirosis, mainly for epidemiological studies, as it is able to test for several serovars that represent different serogroups at once. The principle of the technique is based on the antigen-antibody reaction and detects both IgM and IgG classes of antibodies. The MAT is carried out in two stages. First is screening, in which only the reactive sera in the initial dilution (commonly 1:100) are considered positive and subjected to a second step for titration; in this case; the serum is diluted consecutively in a two-fold ratio and analyzed until the dilution where the serum stops reacting (between 50 and 100% leptospires free under dark field microscopy). In interpreting the results, many laboratories end up determining the positive limit of the test with a titer of 100 (1, 4).

**Figure 1:**
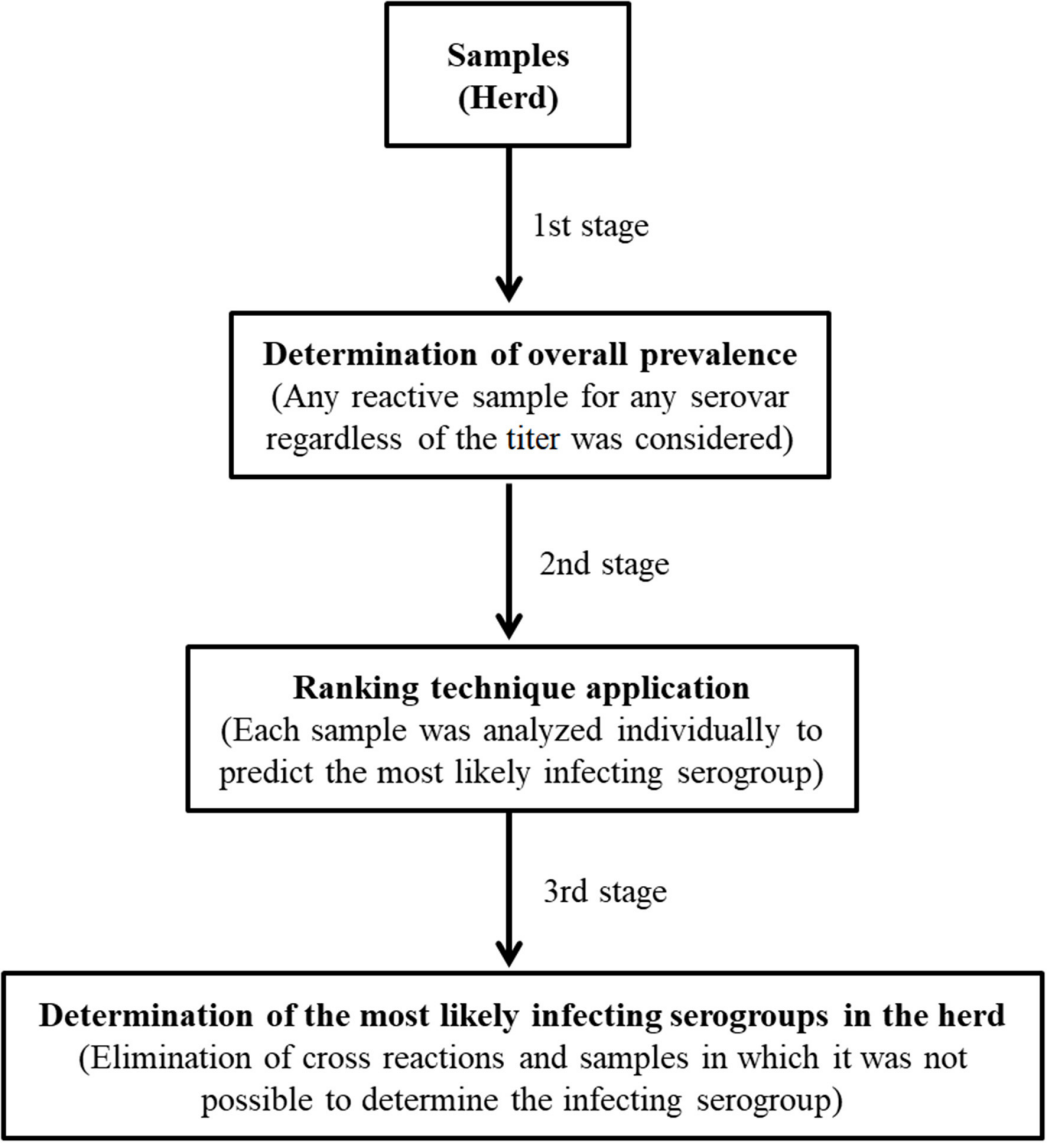
Application of the ranking technique in the interpretation of the results by MAT for a herd.

Using the ranking technique, initially named the most likely serotype, only the reaction for a single serogroup with the highest titer is considered per animal, while other reactions with lower titers for other serogroups are disregarded, as well as samples in which a tie occurs for two or more serogroups with predominant titers (5). A general scheme for how the ranking technique can be used in MAT for a herd is shown in Figure 1. This technique can be very useful for epidemiological studies, as it seems to reduce paradoxical reactions, being used both for herds (5–8) and for humans (9); however, the ability of this technique to identify the presumptive infecting serogroup, namely, the most likely, has not been examined. Thus, the aim of this study was to evaluate the usefulness of the ranking technique for predicting the most likely infecting serogroup with the MAT carried out with serum from experimentally infected hamsters.

## Materials and Methods

This study was carried out from August to October 2020 and approved by the Ethics Committee on Animal Use of the School of Veterinary Medicine and Animal Science (Universidade de São Paulo) – CEUA/FMVZ no. 5724220920. Twelve strains of *Leptospira*, isolated from cattle raised in the Brazilian Amazon (Table 1), were grown in the EMJH medium (Ellinghausen-McCullough-Johnson-Harris) until they reached a concentration of ~2 × 10^8^ leptospires/mL. These samples were chosen because they were isolated from cattle, and most of the laboratory routine tested by us is focused on this species, further considering that it is a species in which cross reactions in serology are observed. In addition, since bovine leptospirosis is a herd disease, the diagnosis of this disease must always be performed at the herd level.

**Table 1:** Strains of leptospires used in this study (10).

**Strain**	**Species**	**Serogroup**
M02/20-52	*L. borgpetersenii*	Sejroe
M02/20-121	*L. borgpetersenii*	Sejroe
M02/20-144	*L. kirschneri*	Autumnalis
M02/20-126	*L. kirschneri*	Grippotyphosa
M02/20-111	*L. kirschneri*	Icterohaemorrhagiae
M02/20-03	*L. santarosai*	Autumnalis
M02/20-18	*L. santarosai*	Tarassovi
M02/20-114	*L. santarosai*	Not determined
M02/20-115	*L. santarosai*	Autumnalis
M02/20-46	*L. noguchii*	Panama
M02/20-155	*L. interrogans*	Canicola
M02/20-136	*L. interrogans*	Icterohaemorrhagiae

Before inoculation of the strains in hamsters (*Mesocricetus auratus*), blood was collected from the animals and subjected to serology using the MAT according to Faine et al. (1) to verify the absence of anti-*Leptospira* spp. antibodies. Then, 0.5 mL of each strain was inoculated intraperitoneally into hamsters weighing between 60 and 100 g for a total of 24 adult animals (2 animals per strain).

The animals were kept under observation for a period of 30 days or until the manifestation of clinical signs of leptospirosis (bristly hair, prostration, photophobia, jaundice, and weight loss), and the presence of these symptoms was the determining factor for carrying out euthanasia, thus avoiding natural death and prolonged stress. In this way, the hamsters were anesthetized, and blood collection was carried out through cardiac puncture. Immediately after, euthanasia and necropsy of the animals were performed, and fragments of the kidneys (1 × 1 cm) were obtained and macerated in sterile Sorensen buffered saline (1:10) and observed directly under dark field microscopy for detection of leptospires. Thus, tissue colonization and renal carriage were confirmed.

The serum samples from the animals were subjected to the MAT by applying a panel with 24 serovars of *Leptospira* spp. used in the routine serological diagnosis of leptospirosis by the laboratory (Supplementary Material 1); moreover, the serum was also tested against the respective strain with which the animal was inoculated (challenge strains). The results were tabulated and analyzed using the ranking technique, considering only the predominant titers found in the MAT for each serum sample, and in this way, stipulating which serogroup was the most likely that infected the animal, discarding the cross reactions with the antigens of the standard MAT panel.

## Results

All animals used in this study, before the inoculation of the *Leptospira* strains, were negative in the MAT, ensuring that after the inoculation, the detected antibodies were obtained only due to the immune response against the inoculated strains. Furthermore, leptospires were detected in the kidneys of all post inoculation animals, demonstrating the ability of the strains to colonize renal tissue and consequently make the animal a carrier. No clinical signs suggestive of leptospirosis or macroscopic lesions on the organs of the animals were observed.

Regarding serology, the ranking technique did not alter the execution of the MAT in any way; it was only applied in the interpretations of MAT results, which were grouped into cases (Table 2). These cases represented possible situations that are commonly observed in the performance of serological studies. It is important to emphasize that the highest titers (800–6,400) were detected against the inoculated strains themselves (challenge strains), meaning there was an immune response from the animals, and any reaction to another serogroup other than that which the inoculated strain belonged was considered a cross reaction.

**Table 2:** Results obtained by serology performed with the serum of animals inoculated with *Leptospira* spp.

**Case**	**Strain**	**Serogroup strain**	**Serology (MAT)**		**Serogroup (ranking technique)**
			**Challenge strain titer**	**Serovar/serogroup titer**	
1	M02/20-126	Grippotyphosa	126: 800	Grip/Grip: 400^*^	Grippotyphosa
	M02/20-46	Panama	46: 1600	Pan/Pan: 800^*^	Panama
	M02/20-18	Tarassovi	18: 800	Tara/Tara: 400^*^	Tarassovi
2	M02/20-52	Sejroe	52: 800	Gua/Sej: 100 Hard/Sej: 400^*^ Hbov/Sej: 400^*^	Sejroe
	M02/20-121	Sejroe	121: 800	Gua/Sej: 100 Hard/Sej: 400^*^ Hbov/Sej: 400^*^	Sejroe
	M02/20-136	Icterohaemorrhagiae	136: 1600	Cope/Ict: 800^*^ Ict/Ict: 200	Icterohaemorrhagiae
3	M02/20-03	Autumnalis	03: 3200	But/Aut: 1600^*^ Cyn/Cyn: 200	Autumnalis
	M02/20-155	Canicola	155: 3200	Can/Can:800^*^ Pan/Pan:400	Canicola
	M02/20-144	Autumnalis	144: 6400	But/Aut: 800^*^ Grip/Grip: 400	Autumnalis
	M02/20-111	Icterohaemorrhagiae	111: 1600	Cop/Ict: 400^*^ Can/Can:100	Icterohaemorrhagiae
	M02/20-115	Autumnalis	115: 3.200	Aut/Aut: 1600^*^ Brat/Brat:200 Pom/Pom: 100	Autumnalis
4	M02/20-114	Not determined	114: 800	Negative	Not determined

*Grip, Grippotyphosa; Pan, Panama; Tara, Tarassovi; Gua, Guaricura; Sej, Sejroe; Hard, Hardjo-Prajitno; Hbov, Hardjo-Bovis; Cope, Copenhageni; Ict, Icterohaemorrhagiae; But, Butembo; Aut, Autumnalis; Cyn, Cynopteri; Can, Canicola; Brat, Bratislava; Pom, Pomona*.

* *Highest titer found and considered the most likely*.

## Discussion

Despite in the literature there are no reports of evaluate the effectiveness of the ranking technique in a controlled way, when the infectious serogroup is known (experimental inoculation), this technique were used in MAT serology for random herds of cattle, buffaloes, sheep, horses and other species (5–8).

First, it is worth noting that the ranking technique, when used, does not negate any sample that was reactive for at least one antigen of the MAT panel, regardless of the titer found, and if there was a tie between antigens from different serogroups, the sample remains positive for leptospirosis and can be considered for calculating the general prevalence of the disease within a herd; however, it is not possible to predict the most likely infecting serogroup for this sample, so it is disregarded only to stipulate the serogroups prevalent in a herd (5–8).

In case 1, the predominant serogroup was the only serogroup detected in the MAT, and no cross reactions were detected since the only serogroups found were the same serogroups to which the challenge strains belonged.

In case 2, reactions were found for more than one antigen, but the antigens belonged to the same serogroup, and even though there was a tie between two antigens with the highest titer (Hard/Hbov), both are in the Sejroe serogroup; in this situation, the predominant serogroup was Sejroe. This phenomenon happens due to the cross reaction that normally occurs between serovars that belong to the same serogroup (1, 2).

In case 3, the animals were reactive for more than a single serogroup, and there was a variation in the titers, with the predominant titer attributed to the most likely serogroup, and the other reactions for the other serogroups disregarded. In addition, the most likely serogroup indicated by the ranking technique was compatible with the serogroup of the *Leptospira* strains inoculated in the respective animal. This seems to be the greatest utility of the ranking technique applied in the MAT for a herd because, if case 3 represented a herd, we would have identified the serogroups Autumnalis, Cynopteri, Canicola, Panama, Grippotyphosa, Icterohaemorrhagiae, Bratislava and Pomona, when in fact the real serogroups circulating in the herd would be Autumnalis, Canicola and Icterohaemorrhagiae; the other serogroups would be overestimated. In this case it is also common occurs a tie for two or more serogroups with predominant titers in a same sample, which would result in the disregard of this sample for the definition of the prevalent serogroups within the herd, however it would continue to be considered reactive for leptospirosis. This situation was not observed in this study.

During the organization and serological classification of leptospires, some serovars that belonged to a serogroup were removed and grouped into other serogroups due to serological affinity, such as what happened with the serovar Butembo, which previously belonged to the serogroup Cynopteri and was later added to the serogroup Autumnalis, remaining today in this serogroup (11). This indicates that antigens from different serogroups can be serologically related, which does not exclude the possibility of cross reaction between them.

Cross reactions between different serogroups usually occur in the MAT during acute cases of the disease since there is an increase in the production of IgM, a circumstance that makes it difficult to interpret the test (1, 12). Nevertheless, in this study, the serology of the animals was performed 30 days after inoculation, when the disease was in the chronic phase (13), and cross reactions were also found. This situation is frequently observed in epidemiological studies, wherein random sampling, it is not possible to establish which phase of the disease the animal is in (5–8).

The identification of acute cases occurs mainly in the presence of clinical signs of the disease (1); however, clinical signs may not be so easily detected, as occurs in cattle infected with strains adapted to them, such as those belonging to the Sejroe serogroup, which silently compromises the reproductive system of these animals (14). Further, different *Leptospira* strains (non-Sejroe) recovered from bovines without observation of clinical signs demonstrates the adaptability that these bacteria may have in the host (15).

We found it interesting to include a *Leptospira* strain in which the serogroup had not been determined, which was represented in case 4, and as a result, the animals were not reactive in the MAT and thus considered negative for leptospirosis; nonetheless, the animals were reactive for the strain that was inoculated and showed renal colonization. Apparently, strain M02/20-114 belongs to a serogroup that is not represented in the MAT panel that was used, which reinforces the importance of adapting the panel of antigens utilized in the MAT, inserting locally isolated strains to increase the sensitivity of the test (2, 7). It should be emphasized that, in cattle, leptospires can be recovered from the urine of negative animals in serology (16), which would be important for an efficient diagnosis the association with other techniques such as PCR, for example.

In conclusion, MAT is a traditional test and are provides a richness of information, therefore its replacement may be difficult to happen. The ranking technique is another way of interpreting the results obtained by MAT, in order to refine the data reducing the occurrence of cross reactions between the serogroups. Thus, we demonstrate how this technique can be useful in the MAT for predicting the most likely infecting serogroup of *Leptospira* and can be applied, especially in epidemiological studies involving herds.

## Data Availability Statement

The raw data supporting the conclusions of this article will be made available by the authors, without undue reservation.

## Ethics Statement

The animal study was reviewed and approved by CEUA FMVZ 5724220920.

## Author Contributions

IG, GS, JC, MC, and AS performed the all laboratory tests. IG and MH performed the interpretation of the results. IG wrote the manuscript. MH accurately reviewed the manuscript. All authors contributed to the article and approved the submitted version.

## Conflict of Interest

The authors declare that the research was conducted in the absence of any commercial or financial relationships that could be construed as a potential conflict of interest.
