# Supplementary material for: Usefulness of the Ranking Technique in the Microscopic Agglutination Test (MAT) to Predict the Most Likely Infecting Serogroup of Leptospira
Source: Front Vet Sci. 2021 Mar 3;8:654034. doi: 10.3389/fvets.2021.654034 (PMC7965942; doi:10.3389/fvets.2021.654034)
Supplement: Supplementary file 1 [file Data_Sheet_1.PDF]

Supplementary material 1

Antigens of *Leptospira* spp. used in our MAT panel applied in the diagnosis of leptospirosis in animals.

Reference strains		
**Species**	**Serogroup**	**Serovar**
L. borgpetersenii	Ballum	Castellonis
	Sejroe	Hardjo-bovis
	Javanica	Javanica
	Tarassovi	Tarassovi
	Celledoni	Whitcombi
L. interrogans	Australis	Australis
	Autumnalis	Autumnalis
	Bataviae	Bataviae
	Australis	Bratislava
	Canicola	Canicola
	Icterohaemorrhagiae	Copenhageni
	Sejroe	Hardjo-prajitno
	Hebdomadis	Hebdomadis
	Pomona	Pomona
	Pyrogenes	Pyrogenes
	Icterohaemorrhagiae	Icterohaemorrhagiae
	Djasiman	Sentot
L. kirschneri	Grippotyphosa	Grippotyphosa
	Autumnalis	Butembo
	Cynopteri	Cynopteri
L. noguchi	Panama	Panama
L. santarosai	Shermani	Shermani
**Autochthonous strains isolated in Brazil**		
**Species**	**Serogroup**	**Serovar**
L. interrogans	Pomona	Pomona (GR6)[1]
L. santarosai	Sejroe	Guaricura[2]

1. Miraglia F, Moreno AM, Gomes CR, Paixão R, Liuson E, Morais ZM, et al. Isolation and characterization of *Lepospira interrogans* form pigs slaughtered in São Paulo State, Brazil. *Braz. J. Microbiol.* (2008) 39:501-507. http://dx.doi.org/10.1590/S1517-83822008000300017

2. Vasconcellos SA, Oliveira JCF, Morais ZM, Baruselli PS, Amaral R, Pinheiro SR, et al. Isolation of *Leptospira santarosai*, serovar Guaricura from buffaloes (*Bubalus bubalis)* in Vale do Ribeira, São Paulo, Brazil. *Braz. J. Microbiol.* (2001) 32:298-300. http://dx.doi.org/10.1590/S1517-83822001000400008
